# Circular RNA RSU1 promotes retinal vascular dysfunction by regulating miR-345-3p/TAZ

**DOI:** 10.1038/s42003-023-05064-x

**Published:** 2023-07-13

**Authors:** Yiting Zhang, Jianping Hu, Xiaoying Qu, Ke Hu

**Affiliations:** 1 The First Affiliated Hospital of Chongqing Medical University, Chongqing Key Laboratory of Ophthalmology, Chongqing Eye Institute, and Chongqing Branch of National Clinical Research Center for Ocular Diseases, Chongqing, China; 2 Department of Ophthalmology, Shanghai Ninth People’s Hospital, Shanghai Jiao Tong University School of Medicine, Shanghai, China; 3 Shanghai Key Laboratory of Orbital Diseases and Ocular Oncology, Shanghai, China; 4 Health Management Center, The First Affiliated Hospital of Chongqing Medical University, Chongqing, China

**Keywords:** Retinal diseases, Genetics research, Angiogenesis

## Abstract

Diabetic mellitus-induced diabetic retinopathy is a significant cause of visual impairment and blindness in adults. Circular RNAs (circRNAs) have been shown to play initial roles in vascular progression. However, the mechanism underlying diabetes mellitus-induced vascular complications remains largely unknown. In circRNA chip experiments, circRSU1 was found to be generally overexpressed in diabetic retinopathy patients. Human retina endothelial cells were stably transfected with lentiviruses carrying a circRSU1 interference plasmid. CircRSU1 downregulation alleviated diabetes mellitus induced retina vascular dysfunction, resulting in decreased vascular endothelial growth factor levels, inflammatory responses and oxidative stress. Mechanistically, we showed that elevated circRSU1 expression upregulated the TAZ levels by sponging miR-345-3p. Downregulation of TAZ reversed the vascular dysfunction that was caused by increased circRSU1 expression under hyperglycaemic conditions. In conclusion, overexpression of circRSU1 promotes vascular dysfunction by sponging miR-345-3p to increase the TAZ levels under diabetic conditions. We provide evidence that circRSU1 is a potential therapeutic target for treating diabetes mellitus-induced vascular dysfunction.

## Abstract (web summary)

The circular RNA circRSU1 is overexpressed in diabetic retinopathy patients and circRSU1 sponges miR-345-3p, increasing TAZ levels, whose downregulation can reverse vascular dysfunction.

## Introduction

Diabetic retinopathy (DR), which is a leading cause of devastating vision loss, is believed to cause blindness in working-age adults. DR is a common microvascular complication of diabetes mellitus (DM)^1^. The worldwide prevalence of diabetes mellitus was estimated to be 463 million in 2019 and is expected to increase to 700 million by 2045. Of these diabetes mellitus patients, 30% have DR^2,3^. Currently, nearly 50% of people living with diabetes do not know that they have the disease^2^. Considering the large number of DR patients, the increasing prevalence, and the severely vision-limiting prognosis, there is an urgent need to develop more effective methods for treating this disease.

Long-term hyperglycemia is considered to be the major risk factor for the development of DR. Retinal microvascular damage that is caused by diabetes mellitus is considered to be the leading cause of vision loss. Retinal vessels are often destroyed in the early stage of diabetes mellitus, retina vessel damage is manifested by pericyte loss, blood-retinal barrier breakdown and capillary cellularity, and it eventually leads to vasogenic edema and nerve tissue damage^4,5^. The mechanism underlying retinal vascular dysfunction in diabetes mellitus patients involves changes in endothelial cell permeability that are caused by advanced growth factors, cytokines and glycation end products^4^. Subsequently, structural damage in endothelial cells and functional modifications in pericytes and astrocytes, all accelerate vascular leakage and incompetence. Retinal vascular remodeling and capillary non-perfusion occur in DR, leading to abnormal neovascularization and ultimately to vision impairment. However, complex molecular mechanisms cannot fully explain the course of the vascular dysfunction, suggesting that other risk factors also play a role. In addition, similar patterns are observed during the pathology of diabetes-induced vascular dysfunction in the retina, glomerulus and vasa neryorum^6^. Understanding the complex cellular and molecular processes that are involved in vascular dysfunction in DR can provide effective treatment options for other vascular complications caused by diabetes mellitus.

Circular RNAs (circRNAs) are endogenous non-coding RNAs that have covalently closed-loop structures and lack a 5′ cap nor a 3′ poly(A) tail^7^. Due to the specific structural elements of circRNAs, they appear to be important, conserved molecules that play roles in biological processes; and additionally they have been shown to be abundant, to be effectively resistant to degradation, and to be able to maintain extraordinary stability^8^. CircRNAs can function as microRNAs (miRNAs) sponges of a particular family, acting as competitive inhibitors that prevent the binding to miRNAs to specific mRNA targets^9^. With the continuous improvement of circRNAs identification technology, studies have shown that circRNAs play important roles in gene expression regulation and protein translation under physiological conditions and during the pathological progression of vascular diseases, metabolic diseases and cancers^10–12^. Previous studies have shown that circ_010383 is significantly downregulated in diabetic nephropathy. circ_010383 inhibits high glucose-induced extracellular matrix deposition and increases TRPC1 expression by sponging miR-135a, thereby inhibiting proteinuria and renal fibrosis^13^. Thus, circRNAs, which are endogenous non-coding RNAs, play roles in initiating diabetes mellitus-induced vascular complications, including DR.

In this study, we identified a potential circRNA, namely, circRSU1 (circ_0006577), that was significantly overexpressed in DR patients and retinal endothelial cells exposed to high-glucose conditions. Mechanistically, elevated circRSU1 expression increased transcriptional coactivator with PDZ-binding motif (TAZ) expression to facilitate endothelial angiogenic function in DR by acting as a sponge for miR-345-3p. Our findings revealed that circRSU1 exacerbated DR progression and may be a novel therapeutic target for DR.

## Results

### Analysis of circRNAs in human retinal endothelial cells

**Fig. 1:**
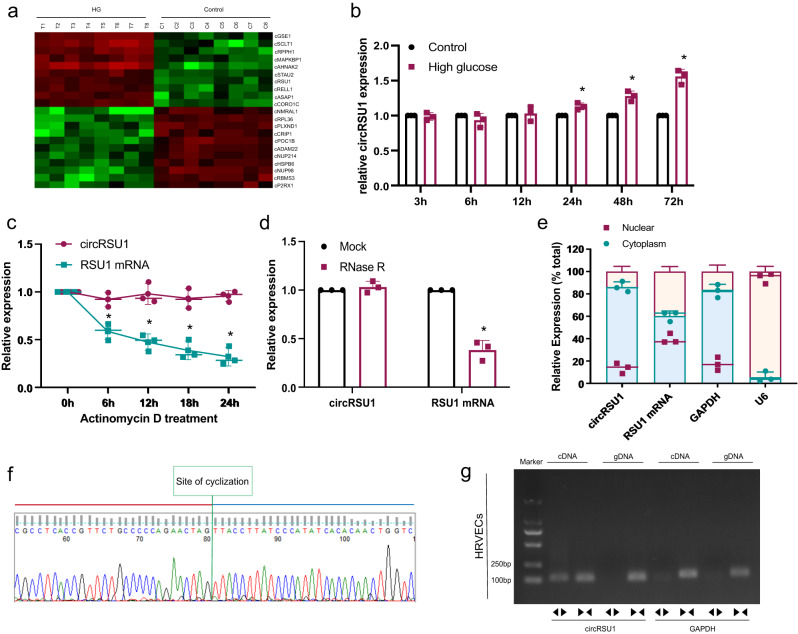
circRSU1-expression pattern in HRVECs. **a** Analysis of the significant circRNAs expression clustering of basal HRVECs and high-glucose HRVECs. **b** circRSU1 expression in HRVECs for a period (3, 6, 12, 24, 48 and 72 h). **c** The amount of circRUS1 and RSU1 mRNA in HRVECs after actinomycin D treatment. **d** After RNase R digestion, qRT-PCR assay was conducted to detect circRSU1-expression level. RSU1 mRNA level was measured as control. **e** circRSU1, RSU1 mRNA, GAPDH (cytoplasm control) and U6 (nuclear control) expression levels were analyzed by qRT-PCR. **f** The sequence obtained from Sanger sequencing matched the sequence of circRSU1 listed in circBase. **g** PCR analysis revealed the presence of circRSU1 through backsplicing. Using divergent primers, circRSU1 was successfully amplified in cDNA samples while showing no amplification in gDNA samples. A negative control with GAPDH was utilized. Unpaired student’s *t*-test, two-way ANOVA test, and Bonferroni test were used for the statistical analyses. *p < 0.05.

Previous studies have shown that retinal endothelial cells play critical roles in the complicated mechanism underlying diabetes mellitus-induced retinal vascular progression^4,6^. Transcriptomic analysis was conducted to identify circRNAs that are expressed by primary human retinal vascular endothelial cells (HRVECs) under baseline conditions (*n* = 8) and high-glucose conditions at different time points (*n* = 4 for 24 h and *n* = 4 for 72 h). Multiple circRNAs were deferentially expressed in the two groups. Hierarchical clustering showed 10 upregulated circRNA candidates and 10 downregulation circRNA candidates in HRVECs, which were identified based on the criteria of |log2 fold-change (FC)| > log25 and -log10 false discovery rate (FDR)  > 2 (Fig. 1a). CircRUS1 was significantly overexpressed in HRVECs under high-glucose conditions (both 24 h and 72 h) with a highly stable upregulation level from 24 h and lasted more than 72 h (Fig. 1b). CircRSU1 had a looped structure with a length of 489 bp and its gene is located on chr10: 16794537–16824083 in the human genome (hsa_circ_0006577) according to circBase^14^. circRSU1 was stable with a half-life of more than 24 h after actinomycin D treatment. In contrast, linear RSU1 mRNA had a half-life of less than 6 h (Fig. 1c). Furthermore, circRSU1 was more resistant to RNase R treatment than linear RSU1 mRNA in HRVECs (Fig. 1d). Finally, we observed high expression of circRSU1 in the cytoplasm of HRVECs using qRT-PCR (Fig. 1e). The amplified product of circRSU1 was subjected to sequencing. The sequencing analysis revealed a complete match between the obtained sequence and the known sequence of circRSU1 listed in circBase (Fig. 1f and Supplementary Fig. 1). The results demonstrated a sequence consistency, confirming the accurate identification of circHIPK3. In order to eliminate the possibility of genomic rearrangement of the host gene, we developed specific primers for RSU1 mRNA and circRSU1 in opposite orientations. We extracted cDNA and genomic DNA from HRVECs and performed agarose gel electrophoresis to analyze them. Through this analysis, we successfully amplified circRSU1 using the primers designed for circRSU1 in the cDNA samples, while no amplification was observed in the genomic DNA samples. This confirmed the existence of circularized RSU1 exons and ruled out the presence of trans-splicing products (Fig. 1g).

Moreover, the demographic and clinical characteristics of diabetes mellitus patients with or without DR are summarized in Table 1. Compared with participants without DR, patients with DR were more likely to have higher BMI, longer duration of diabetes mellitus, and higher expression levels of circRSU1 and vascular endothelial growth factor (VEGF) compared with patients without DR. Cox regression analysis showed that the BMI, diabetes duration and VEGF level were independent prognostic factors for DR patients with high circRSU1 levels (Table 2). These results suggested that circRSU1 may exacerbate DR progression in patients and that circRSU1 may be a major driver of high-glucose-induced retinal vascular endothelial cell dysfunction during DR progression.

**Table 1:** Clinical characteristics of patients with diabetes mellitus and DR.

Characteristics	DM (*n* = 53)	DR (*n* = 50)	*P*
CircRSU1 (log transformed)	1.31 ± 0.24	1.61 ± 0.37	<0.001*
Age (years)	54.49 ± 5.49	56.84 ± 5.63	0.182
Male/female	26/27	23/27	0.098
Body mass index, BMI (kg/m^2^)	23.38 ± 2.13	23.93 ± 2.04	<0.001*
Diabetes duration (years)	5.34 ± 2.01	8.98 ± 2.37	0.028*
Family history	11 (20.75%)	12 (24%)	0.136
Fasting glucose (mmol/L)	8.32 ± 1.36	8.87 ± 1.32	0.051
HbA_1c_	7.95 ± 1.19	8.38 ± 1.23	0.209
VEGF (pg/mL)	47.74 ± 0.97	58.22 ± 5.39	<0.001*

*p < 0.05 was regarded as statistically significant.

**Table 2:** Univariate and multivariate analyses of factors associated with circRSU1.

Level of circRSU1	Univariate analysis		Multivariate analysis	
	*P*	HR	*P*	95% CI
Age, years (≥56 vs. <56)	0.552			
Gender (Male vs. Female)	0.383			
BMI (≥24 vs. <24)	0.004*	1.417	<0.001*	1.232–2.091
Diabetes duration, years (≥9 vs. <9)	0.016*	0.753	0.113*	0.631–1.05
Family history (Yes vs. No)	0.218			
Fasting glucose, mmol/L (≥8.8 vs. <8.8)	0.702			
HbA_1c_ (≥8.3 vs. <8.3)	0.216			
VEGF, pg/mL (≥58 vs. <58)	<0.001*	0.18	<0.001*	0.134–0.224

*p < 0.05 was regarded as statistically significant.

### circRSU1 promoted the proliferation and migration of human retinal endothelial cells under hyperglycaemic conditions

**Fig. 2:**
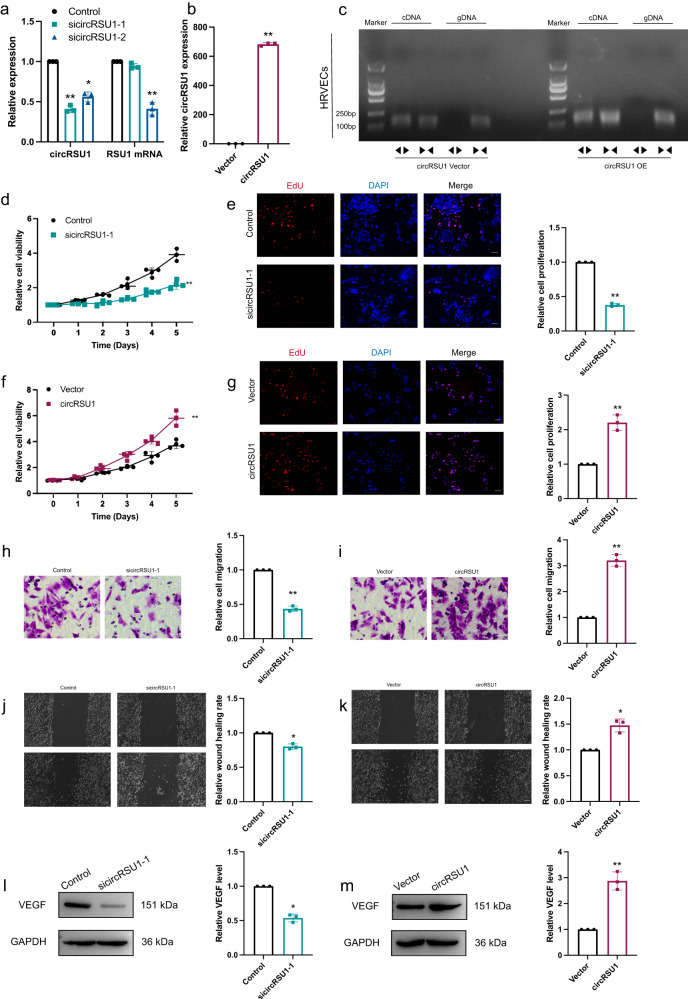
circRSU1 promoted DR progression. **a**, **b** The efficiency of circRSU1 interference was determined by qRT-PCR. **c** PCR analysis revealed the presence of circRSU1 overexpression (OE) through backsplicing. A negative control with circRSU1 vector was utilized. **d**–**g**. MTT assay and EdU staining were performed to detect the proliferation of HRVECs (scale bar = 100 μm). **h**, **i** Transwell invasion assay was used to detect the invasion ability of HRVECs (scale bar = 20 μm). **j**, **k**. Wound-healing migration assay was performed to detect the migration ability of HRVECs (scale bar = 100 μm). **l**, **m** Western blot was used to analyze VEGF expression. GAPDH was used as a negative control. Unpaired student’s *t*-test, Mann–Whitney *U* test and one-way ANOVA test were used for the statistical analyses. *p < 0.05,**p < 0.01.

To explore the function of circRSU1 in detail, we designed two small interfering RNAs (siRNAs) to silence circRUS1 (sicircRSU1-1 and sicricRSU1-2). CircRSU1 levels were significantly downregulated by sicircRSU1-1 in HRVECs (Fig. 2a). Using the pLC5 plasmid vector, circRSU1 overexpression was successfully achieved in HRVECs (Fig. 2b). To ensure the presence of overexpressed circularized RSU1, we used the convergent primers for RSU1 mRNA and divergent primers for circRSU1. Analysis of cDNA and genomic DNA from HRVECs using agarose gel electrophoresis revealed specific amplification of overexpressed circRSU1 with divergent primers in cDNA only (Fig. 2c). MTT assays and EdU incorporation assays revealed that circRSU1 knockdown decreased HRVECs viability and proliferation, and these effects were reversed by circRSU1 overexpression (Fig. 2d–g). Transwell invasion assays and wound-healing migration assays revealed that the migration and invasion of HRVECs were inhibited after circRSU1 downregulation, while circRSU1 overexpression increased HRVEC migration and invasion (Fig. 2h–k). We then performed western blotting analysis. The results revealed that decreased circRSU1expression resulted in VEGF downregulation, while increased circRSU1 expression resulted in VEGF upregulation (Fig. 2l, m). Overall, overexpression of circRSU1 accelerated the proliferation, migration and invasion of HRVECs and facilitated endothelial angiogenesis under high-glucose conditions.

### circRSU1 upregulated TAZ levels by acting as a miR-345-3p sponge

**Fig. 3:**
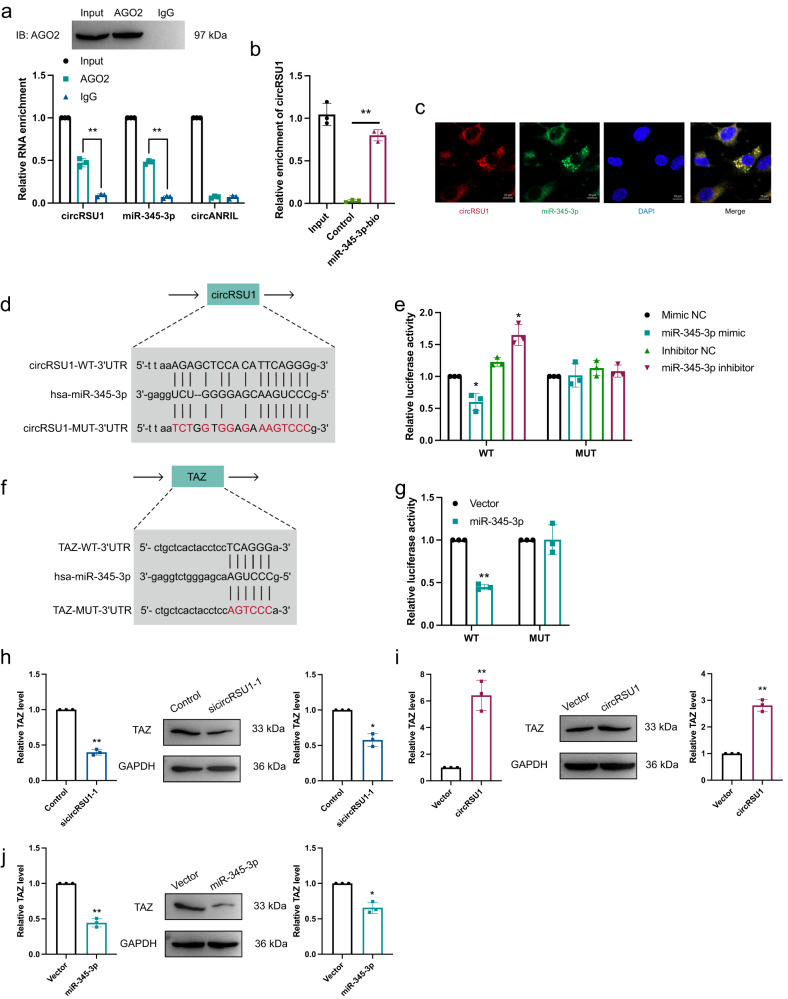
circRSU1 acted as a sponge of miR-345-3p. **a** RIP experiments were carried out using AGO2 or IgG antibody. **b** RNA pull-down analysis using biotinylated miR-345-3p mimic or mimic NC probe, and the precipitated RNA was analyzed by qRT-PCR. **c** circRSU1 and miR-345-3p were detected by FISH assay (scale bar = 10 μm). **d** Schematic diagram showed the binding sites between circRSU1 and miR-345-3p. **e** The luciferase activity of circRSU1 in HEK-293 T cells after co-transfection with miR-345-3p. **f** Schematic diagram showed the binding sites between miR-345-3p and TAZ. **g** The luciferase activity of miR-345-3p in HEK-293 T cells after co-transfection with TAZ. **h**–**j** qRT-PCR was performed to analyze TAZ expression in different treatments. Unpaired student’s *t*-test and Mann–Whitney *U* test were used for the statistical analyses. *p < 0.05, **p < 0.01.

CircRNAs participate in biological processes mainly through their function as miRNA sponges^15^. Additionally, circRSU1 was highly expressed in the cytoplasm of HRVECs. Therefore, we hypothesized that circRSU1 may perform an efficient function in the progression of vascular dysfunction by sponging specific miRNAs. First, we used TargetScan and miRanda to predict potential miRNA targets of circRSU1, and miR-345-3p was identified in both of these databases. To analyze the interaction between circRSU1 and miR-345-3p, we performed an RNA immunoprecipitation assay (RIP) in HRVECs using an anti-Argonaute 2 (AGO2) antibody. The results showed that circRSU1 and miR-345-3p were significantly enriched by the anti-AGO2 antibody but not IgG. In contrast, circANRIL, which is a circRNA that does not bind to AGO2, was not found to be enriched (Fig. 3a). RNA pull-down assays showed the miR-345-3p probe significantly enriched circRSU1 in HRVEC cells compared to the control probe (Fig. 3b). The results indicated that circRUS1 may function as a sponge for miR-345-3p. According to FISH analysis, we observed that both circRSU1 and miR-345-3p were highly expressed in the cytoplasm of HRVECs, and they were obviously co-localized (Fig. 3c). Subsequently, luciferase reporter assays were performed to further analyze the interaction between circRSU1 and miR-345-3p (Fig. 3d). The wild-type circRSU1 sequence that contains miR-345-3p binding sites and mutant sequence were cloned into psiCHECK2, and then the vectors were transfected into HEK-293 T cells. Transfection with the miR-345-3p mimics and the wild-type sequence resulted in significantly reduced the luciferase reporter activity compared with the mutant sequence, while transfection with the miR-345-3p inhibitor and the wild-type sequence resulted in significantly increased luciferase reporter activity compared with the mutant sequence (Fig. 3e).

Previous studies indicated that miRNAs could inhibit certain mRNAs in pathological progression^9^. We speculated that circRSU1 accelerated vascular dysfunction by protecting related mRNAs from downregulation by miR-345-3p. Using the TargetScan database, we identified a potential target of miR-345-3p, namely, TAZ (Fig. 3f). TAZ has been reported to promote angiogenesis and contribute to insulin resistance^16,17^. Thus, we performed luciferase reporter assay to confirm that circRSU1 promoted vascular dysfunction by protecting TAZ from miR-345-3p-mediated inhibition. Luciferase reporter vectors carrying wild-type or mutant TAZ sequences were transfected into HEK-293 T cells. Compared with the mutant type TAZ sequences, the luciferase reporter activity was significantly reduced in cells transfected with the wild-type TAZ sequences (Fig. 3g). Moreover, the level of TAZ was significantly decreased after circRSU1 downregulation or miR-345-3p upregulation. Furthermore, the TAZ expression level was increased after circRSU1 overexpression (Fig. 3h–j). Together, these results showed that circRSU1 upregulates TAZ by directly binding to miR-345-3p in HRVECs.

### Elevated TAZ promoted the proliferation, migration and invasion of HRVECs

**Fig. 4:**
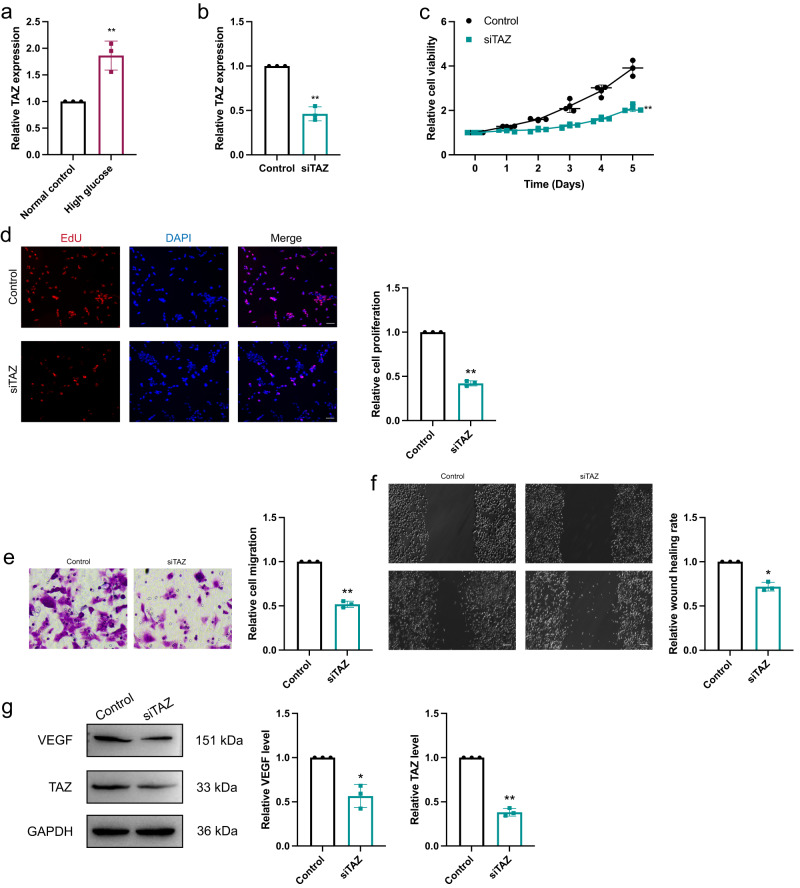
TAZ promoted DR progression. **a**, **b** The efficiency of TAZ interference was determined by qRT-PCR. **c**, **d** MTT assay and EdU staining were performed to detect the proliferation of HRVECs (scale bar = 100 μm). **e** Transwell invasion assay was used to detect the invasion ability of HRVECs (scale bar = 20 μm). **f** Wound-healing migration assay was performed to detect the migration ability of HRVECs (scale bar=100 μm). **g** Western blot was used to analyz VEGF and TAZ expression. GAPDH was used as a negative control. Unpaired student’s *t*-test, Mann–Whitney *U* test and one-way ANOVA test were used for the statistical analyses. *p < 0.05, **p < 0.01.

The potential role of TAZ as a miR-345-3p target in circRSU1-induced HRVECs dysfunction was further explored in HRVECs. According to qRT-PCR, we observed that the TAZ levels were significantly upregulated in HRVECs under high-glucose conditions compared with baseline conditions (Fig. 4a). After transfection with the siTAZ vector, transfection efficiency was measured by qRT-PCR (Fig. 4b). MTT and EdU incorporation assays revealed that the cell viability was inhibited after TAZ knockdown (Fig. 4c, d). Wound-healing migration and transwell invasion assays showed that TAZ downregulation decreased the cell migration and invasion of HRVECs (Fig. 4e, f). Previous studies have reported that TAZ activates multiple pathways during vascular disease progression. We found that TAZ downregulation decreased VEGF expression levels (Fig. 4g). These results indicate that elevated TAZ promotes HRVECs proliferation, migration and invasion.

### TAZ downregulation reversed circRSU1-induced HRVECs dysfunction

**Fig. 5:**
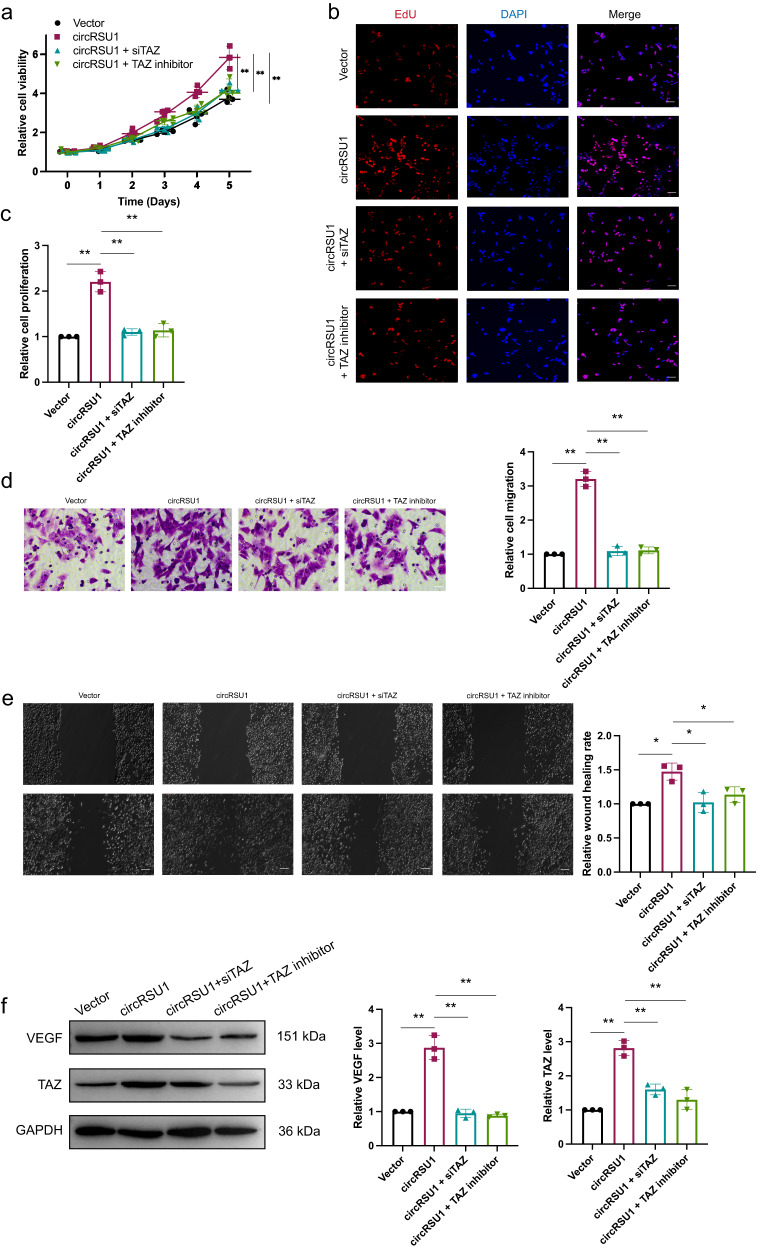
Downregulated TAZ reversed circRSU1-induced HRVECs dysfunction. **a**–**c** MTT assay and EdU staining were performed to detect the proliferation in HRVECs treated with vector, circRSU1, circRSU1 + siTAZ, circRSU1 + TAZ inhibitor (scale bar = 100 μm). **d**, **e**. Transwell invasion assay and wound-healing migration assay were used to detect the invasion ability in HRVECs (scale bar **d** = 20 μm, **e** = 100 μm). **f** Western blot was used to analyze VEGF and TAZ expression in different treatments. GAPDH was used as a negative control. Unpaired student’s t-test and one-way ANOVA test were used for the statistical analyses. *p < 0.05, **p < 0.01.

To further validate the role of circRSU1 in HRVECs progression by sponging miR-345-3p sponge, rescue experiments were conducted. MTT, EdU, wound-healing migration and transwell invasion assays were performed in HRVECs that were transfected with siTAZ and a TAZ inhibitor. The results revealed that the circRSU1-induced increases in viability, proliferation, migration and invasion were inhibited by TAZ inhibition (Fig. 5a–e). Moreover, the high VEGF level induced by circRSU1 overexpression was also reversed by TAZ downregulation (Fig. 5f). These results indicated that TAZ inhibition reversed circRSU1-induced retinal vascular dysfunction in vitro.

### circRUS1 regulated diabetes mellitus-induced retinal vascular dysfunction in vivo

**Fig. 6:**
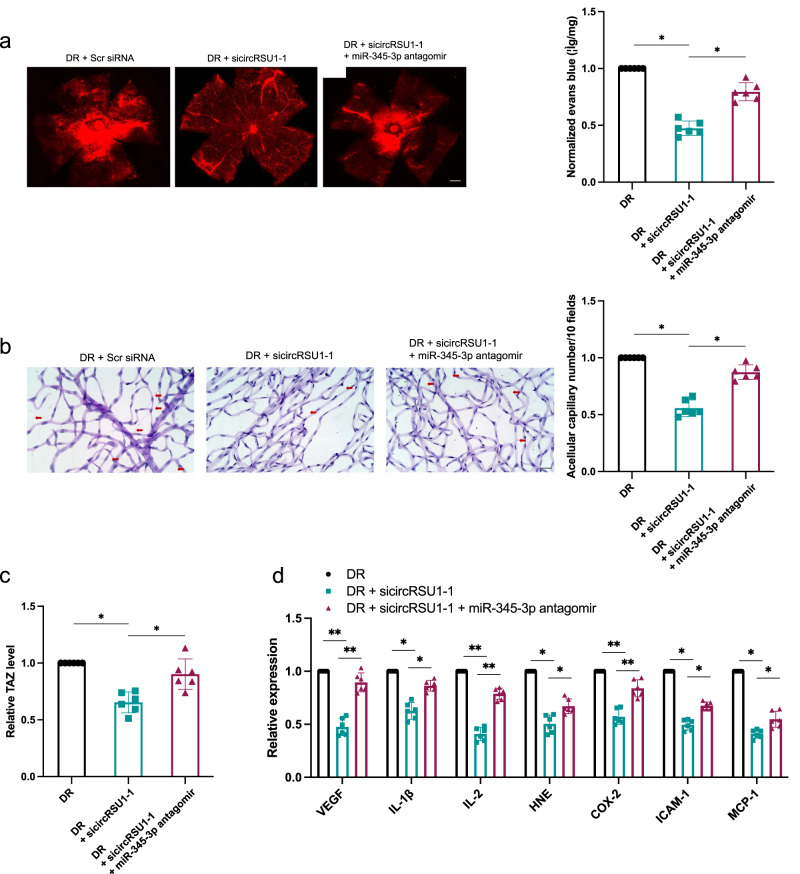
circRSU1 correlated with retina vascular dysfunction in DR. **a** The fluorescence microscope was used to detect Evans blue leakage in DR model rats (scale bar = 100 μm). **b** Retinal trypsin digestion assay was conducted to measure conditions of acellular capillaries in the in DR model rats (scale bar = 50 μm). **c**, **d** Elisa assay was conducted to detect the expression of VEGF, IL-1β, IL-2, HNE, COX-2, ICAM-1 and MCP-1. *N* = 6 eyes in each group. Kruskal–Wallis test followed by the post hoc Bonferroni test was used for the statistical analyses. *p < 0.05, **p < 0.01.

The potential role of circRSU1 in hyperglycemia-induced retinal capillary degeneration and retinal vascular dysfunction was further explored in diabetic rat models. According to the Evans blue leakage assay, circRSU1 downregulation alleviated DR-induced retinal vascular leakage, while these effects were blocked by miR-345-3p inhibition (Fig. 6a). Retinal trypsin digestion revealed that circRSU1 downregulation significantly inhibited the hyperglycemia-induced increase in the number of acellular capillaries, whereas mir-345-3p inhibition increased retinal vascular degeneration (Fig. 6b). We also confirmed that the TAZ levels were downregulated in the sicircRSU1 group, but upregulated after miR-345-3p inhibition (Fig. 6c). Inflammatory cytokines are also responsible for promoting diabetes mellitus-related vascular complications. CircRSU1 downregulation resulted in high levels of VEGF, interleukin-1β (IL-1β), IL-2, 4-Hydroxynonenal (HNE), cyclooxygenases-2 (cox-2), intercellular adhesion molecule-1 (ICAM-1) and monocyte chemoattractant protein-1 (MCP-1), and these levels were reduced by miR-345-3p downregulation (Fig. 6d). Therefore, the results indicated that miR-345-3p knockdown could reverse the effect of circRSU1 downregulation on DR-induced retinal vascular dysfunction.

## Discussion

circRNAs account for a large proportion of RNAs and form covalently closed continuous loop structural; circRNAs possibly play important functional roles in humans^18^. Compared with linear RNAs, circRNAs have been found to be more abundant, conserved and stable in cells. In addition to their potentially critical role in gene regulation, circRNAs may represent a strategy for targeted therapy. Although the therapeutic potential of circRNAs has received increasing attention in laboratories and the clinic, few studies have revealed their mechanism of action in DR-induced retinal vascular dysfunction. Therefore, we hypothesize that circRNAs play major roles in diabetes mellitus-induced vascular complications. In this study, we confirmed that circRSU1 promotes endothelial angiogenesis by regulating retinal vascular endothelial cells and retinal microvascular function. Elevated circRSU1 exacerbates DR progression, which positively correlates with poor prognosis in DR patients. The mechanism was further shown to be that circRSU1 downregulates miR-345-3p levels by acting as a sponge, resulting in activation of the TAZ and VGEF pathway. The present study advances research on the roles of non-coding RNAs in diabetic vascular complications.

Diabetes mellitus-induces retinal vascular dysfunction is thought to be the primary cause of blindness. Endothelial cells tend to be the main target in diabetes mellitus-induced vascular complications, and these complications are triggered by a number of factors including VEGF, inflammation, oxidative stress, endothelial nitric oxide synthase, and endoplasmic reticulum stress^19^. Vascular homeostasis balances insulin levels and stimulates glucose transport to regulate glucose metabolism in the endothelial cells^20^. Previous studies have identified therapeutic strategies for protecting endothelial cells from hyperglycemia, and circRNAs have been shown to be effective in regulating pathways that maintain vascular homeostasis^21,22^. HRVECs cultured under high-glucose conditions mimic the status of retinal endothelial cells in diabetic rat models and diabetes mellitus patients, and the upregulation of circRSU1 expression observed in these HRVECs supported the association of circRSU1 with endothelial dysfunction under hyperglycemia conditions. Under diabetic conditions, endothelial dysfunction is the main factor that causes vascular complications, and it is also the main manifestation of diabetic microvascular complications^23^. We observed a similar circRSU1-expression pattern in HRVECs. Downregulation of circRSU1 attenuates the progression of HRVECs. High-glucose conditions influence the endothelial cell phenotype by activating inflammatory and reactive oxygen species intracellular signaling pathways, affecting vascular homeostasis and accelerating vascular modification^24^. circRSU1 silencing can inhibit retinal vascular leakage and decrease VEGF levels, the inflammatory response and oxidative stress.

Accumulating evidence suggests that circRNAs participate in diverse sets of biological processes, including by sponging miRNAs and regulating RNA transcription and protein translation. CircRNAs can act as specific miRNA sponges and thus act as competitive inhibitors to inhibit the binding of miRNAs to their mRNA targets. The present study shows that circRSU1 acts as a sponge for miR-345-3p and co-localized in the cytoplasm of HRVECs. MiRNAs repress the translation of mRNAs by directly binding to the complementary base pairs of target sites in mRNA 3’-UTRs^25^. According to the miRNA target prediction results, TAZ was found to be a potential target gene of miR-345-3p. TAZ is thought to promote cell proliferation/survival and was therefore selected for the following studies. These results demonstrate that circRSU1 functions as a sponge of miR-345-3p to contribute to diabetes mellitus-induced vascular dysfunction through the TAZ signaling pathway.

TAZ and yes-associated protein (YAP) are related transcriptional coactivators that are regulated by the Hippo pathway and play major roles in endothelial cells via the regulation of behaviors, proliferation, metabolism and junction assembly^26,27^. Endothelial-specific deletion of YAP/TAZ in mice leads to severe morphogenic defects, disrupted vascular barrier integrity and stability, and consequent hemorrhage in growing retina vessels^28^. YAP/TAZ has been shown to be the central mediator of VEGF mediated angiogenic development via a specific transcriptional program that is important for vascular tip cell migration and vascular barrier formation and maturation^26,29^. The present study reveals that circRSU1 acts as a miR-345-3p sponge to reduce TAZ inhibition, leading to TAZ activation. Elevated TAZ levels induce vascular dysfunction by regulating endothelial cell behavior under diabetic conditions. These results indicate that circRSU1 and miR-345-3p participate in the clinical pathogenesis of diabetic retinopathy. This regulatory mechanism provides a potential approach for the treatment of diabetic vascular complications.

We identified a potential circRNA (circRSU1, circ_0006577) that is elevated in DR patients, HRVECs cultured under diabetic conditions and diabetic rat models. Overexpressed circRSU1 positively correlates with diabetes mellitus-induced vascular progression. The results in the present study elucidate the potential mechanism by which circRNAs regulate the progression of vascular function, further suggesting that the circRSU1/miR-345-3p/TAZ signaling pathway may be a potential therapeutic target for diabetes mellitus-induced vascular complications.

## Methods

### Patients and samples

A total of 103 diabetes mellitus (type 2) patients were recruited according to the criteria from the American Diabetes Association^30^. Plasma samples were collected from blood of all the patients, and the patients were classified as diabetes mellitus patients without DR (*n* = 53) and diabetes mellitus patients with DR (*n* = 50). The medical files of all the patients were reviewed by trained certified doctors to collect relevant medical information. CircRNAs levels and VEGF serum levels were measured in this study. The study protocol was approved by the Ethics Committee of the First Affiliated Hospital of Chongqing Medical University and performed in strict accordance with the Declaration of Helsinki. Written informed consent was obtained from all participants.

### Rat diabetic model and intravitreal injection

Male Sprague-Dawley rats (80–100 g) were obtained from SLAC Laboratory Animal Co., Ltd (Shanghai, China) and were used to establish a diabetic animal models. All the animals were house under a 12-h light/dark cycle and received an intraperitoneal injection of streptozotocin (STZ, 65 mg/kg, Sigma) . Fasting blood glucose was measured every week for three months. Rats with blood glucose levels greater than 11.1 mmol/L were considered to have diabetes. Lentivirus carrying scramble siRNA or sicircRSU1-1 was intravitreally injected at 1 × 10^10^ TU/total (2 μL/eye) with a 33-gauge needle at the beginning of diabetes establishment. The TAZ overexpression vector and scramble vector were injected 10 days before diabetes establishment. Then, we performed monthly intravitreal injections to increase the effect. After the sacrifice, the retinas of the diabetic model rats were harvested for biochemical assays. All animal experiments were approved by the Animal Ethics Committee of the First Affiliated Hospital of Chongqing Medical University and met the standards of the Association for Research in Vision and Ophthalmology Statement for Use of Animals in Ophthalmic and Vision Research.

### Cell culture and transfection

Primary human retinal vascular endothelial cells (HRVECs) were obtained from Cell Systems (Kirkland, WA) and cultured in EGM2-MV medium supplemented with 5% of fetal bovine serum (Gibco, Grand Island, NY) at 37 °C in 5% CO_2_. To establish a high-glucose cell culture model, cells were cultured in high-glucose DMEM supplemented with 10% FBS, and the glucose concentration was adjusted to 30 mmol/L. Full-length circRSU1 was amplified by PCR and cloned into pLC5 overexpression plasmids. The primers for vector sequencing were as follows: pC5-seqF: TGTGAATTTGACCCTTAAGA. The circRSU1 primers for qRT-PCR were as follows: F2:AGGGATAACGACCTGATCTC, R2:TGTGATATGGGATAAGGTAACTAG. Synthesized small interfering RNAs (siRNA, Sigma, St Louis, MO) targeting circRSU1 (sicircRSU1-1 F: GAACTAGTTACCTTATCCC; sicircRSU1-2 F: CCCAGAACTAGTTACCTTA) were transiently transfected into cells with Lipofectamine 2000 reagent (Invitrogen, Carlsbad, CA, USA) following the manufacturer’s instructions.

### Actinomycin D treatment and ribonuclease R (RNase R) treatment

For actinomycin D treatment, 2 mg/ml actinomycin D or dimethylsulfoxide (Sigma-Aldrich, St. Louis, MO, USA) as a negative control was added into the cell culture medium. For RNase R treatment, about 2 μg of RNAs were incubated for 30 min at 37 °C with or without 3 U/μg RNase R according to the manufacturer’s instruction. Then the RNAs were purified with the RNeasy MinElute Cleanup Kit (Qiagen).

### RNA extraction and quantitative reverse transcription-PCR (qRT-PCR)

Total RNA was extracted using TRIzol reagent (Invitrogen, Carlsbad, CA, USA). The cytoplasmic and nuclear fractions were extracted using NE-PER Cytoplasmic and Nuclear Extraction Reagents (Thermo Scientific). The miRNA was reversed transcribed using M-MLV Reverse Transcriptase (Promega, Madison, WI, USA) and cDNA was synthesized by PrimeScript RT reagent kit (Promega, Madison, WI, USA). The qRT-PCR was performed using SYBER Green PCR Master mix (Takara Bio, Inc.,Shiga, Japan). All the primer sequences are listed in Supplementary Table 1. Gene expression level was calculated with 2-ΔΔCt method.

### Western blot assays and enzyme-linked immunosorbent assay (ELISA)

Proteins were isolated from Cells using cold RIPA buffer (Beyotime, Haimen, China). After electrophoresis, proteins were transferred onto PVDF membranes (Millipore, Bedford, MA). Then the stripes were blocked with 5% nonfat milk for 2 h, and incubated overnight at 4 °C with the following primary antibody, GAPDH (1:1000, M00227-5, BOSTER), VEGF (1:1000, Abcam) and TAZ (1:500, Abcam). After washing, respective secondary antibodies (1:5000, Abcam) were applied for 2 h at room temperature. The protein bands were visualized using BeyoECL Moon (Beyotime).

The concentrations of VEGF, IL-1β, IL-2, HNE, COX-2, ICAM-1 and MCP-1 in the culture medium were analyzed by commercially available ELISA Kit (R&D Systems, Minneapolis, MN and Abcam).

### Luciferase reporter assay

The wild-type and mutant circRSU1 sequences were synthesized by the whole gene synthesis method, and the target fragments were ligated into the psiCHECK2 vector with XhoI and NotI, respectively. The primer for the vector was as follows : psiCHECK2-F: ATGGGTAAGTACATCAAGAG. The psiCHECK2-circRSU1-WT (TTAAAGAGCTCCACATTCAGGGG), psiCHECK2-circRSU1-MUT (TTAATCTGGTGGAGAAAGTCCCG), miR-345-3p mimics, and miR-345-3p NC were co-transfected into cells. After 24 h, relative luciferase activity was measured by a dual-luciferase reporter kit (Promega, Madison, WI, USA). The psiCHECK2-TAZ-WT 3′-UTR or psiCHECK2-TAZ-MUT 3′-UTR was co-transfected with miR-345-3p mimics or miR-345-3p NC. After transfection for 48 h later, luciferase activity was measured by the Dual-Luciferase Reporter Assay System (Promega).

### RNA fluorescence in situ hybridization (FISH)

RNA-FISH was performed using a Cy3-labeled RNA probe for circRSU1 and a FITC-labeled RNA probe for miR-345-3p. The specific probes were as follows: circRSU1 (5′–3′ CY3 Labeled): TGGGATAAGGTAACTAGTTCTGGGG, miR-345-3p (5′–3′ FITC Labeled): CTCCAGACCCCTCGTTCAGG. Then signals were then detected with a fluorescence in situ hybridization kit (RiboBio) according to the manufacturer’s protocol. The cell nuclei were counterstained with DAPI. Fluorescence images were captured using a Zeiss LSM 800 microscope.

### RNA immunoprecipitation assay (RIP) and biotin-labeled RNA capture

RIP assays were performed with the Magna RIP RNA-Binding Protein Immunoprecipitation Kit (Millipore) following the manufacturer’s instructions. Cells were washed in PBS, lysed in co-IP buffer, and then sonicated and incubated for 3 h at 4 °C. The probe-streptavidin-dynabeads were added, and the mixtures were incubated at 30 °C for 12 h. Next, lysis buffer and proteinase K were added. RNA was extracted according to the TRIzol Reagent and then subjected to qRT-PCR analysis.

MiRNA pull-down assays were performed by transfecting biotinylated miR-345-3p or control probe (Geneseed, Guangzhou, China) into HRVECs with Lipofectamine 8000 (Beyotime, China). The HRVECs lysates were precleared by centrifugation. Then, the remaining lysates and M-280 streptavidin magnetic beads (Invitrogen, USA) were incubated overnight at 4 °C. The biotin-coupled RNA complex bound to the beads was purified using TRIzol reagent. qRT-PCR was conducted to analyze circRSU1 levels in bound fractions.

### MTT assay, EdU staining, wound-healing migration, cell migration assays and Bioinformatics analysis

3-(4, 5-dimethylthiazol-2-yl)-2, 5-diphenyl-tetrazolium-bromide assay (MTT) was used to determined cell viability. The cells were cultured in 96-well plates at a density of 1 × 103 cells per well for 5 days and counted daily. The absorbance value was determined at 490 nm, and the cell proliferation rates were measured by cell growth curve.

EdU (5-ethynyl-2′-deoxyuridine) DNA Cell Proliferation Kit (RiboBio, Guangzhou, China) was used for cell proliferation analysis. Cells were grown on coverslips and incubated with 50 mM EdU for 2 h. After washing and fixing, the cells were incubated with Apollo Dye Solution to stain the proliferating cells. Counterstain was performed using Hoechst (1:1000). Images were randomly captured from six fields (Nikon 80i, Nikon Corporation, Tokyo, Japan).

Cells were plated in 6-well plates and scratched with sterile 10 μl pipette tip to create a wound. After washing, medium without serum was added to the well. Images were taken at 72 h from at least six fields.

The cell migration ability was determined by Matrigel-coated transwell inserts. Cells were plated to Matrigel-coated inserts and allowed to invade through the Transwell plates. The cells attached to the other side were fixed, stained and counted. The number of invading cells was averaged from at least six randomly chosen fields.

### Bioinformatics analysis

We used miRanda and TargetScan databases to predict potential targets of circRSU1, resulting in hundreds of overlapping miRNAs. From this pool, we focused on 36 miRNAs associated with human diseases. Among them, miR-345-3p stood out due to its expression in HUVECs and its reported ability to attenuate growth inhibition, inflammation, and apoptosis in endothelial cells. Through dual-luciferase reporter assays, RIP, and FISH, we confirmed the direct interaction between circRSU1 and miR-345-3p. Additionally, using ENCORI (http://starbase.sysu.edu.cn/) and TargetScan, we predicted TAZ as a potential target of miR-345-3p. TAZ is known for its involvement in angiogenesis and insulin resistance.

Based on these findings, we hypothesized that circRSU1 may regulate vascular dysfunction by modulating the miR-345-3p/TAZ axis. Subsequent experiments were conducted to further elucidate this regulatory mechanism.

### Retinal trypsin digestion assay

The animal eyes were fixed in 4% paraformaldehyde for 24 h and then cut into 4 fragments to remove the retina. The retinas were incubated at 37 °C for 3 h with 3% trypsin. After fixing and washing, the retinal tissues were stained with periodic acid-Schiff and haematoxylin, observed on the slides and photographed.

### Evans blue dye extravasation assay

Jugular vein injection of Evans Blue dye was performed over 10 s. After a 2-h cycle of dye circulation in animals, cold PBS was transcardially perfused followed by 4% paraformaldehyde. Then, the cornea, lens, and vitreous humor of the rat’s eyes were removed. The sclera and retina were fixed with 4% paraformaldehyde. Formamide was added to the samples and incubated overnight at 78 °C. The absorbance of Evans blue dye was measured at 620 nm and 740 nm and compared with that in the samples without formamide.

### Statistics and reproducibility

All the statistical analyses were performed using SPSS software version 23.0 (SPSS, Inc., Chicago, IL, USA). The data are presented as the mean ± standard deviation (SD). Student’s *t*-test, chi-squared test, Mann–Whitney *U* test, one-way or two-way ANOVA test, and Kruskal–Wallis test were performed for comparisons. Independent risk factors were analyzed by Cox’s proportional hazard regression model. *P* < 0.05 was considered statistically significant.

### Reporting summary

Further information on research design is available in the Nature Portfolio Reporting Summary linked to this article.

## Supplementary information

Supplementary materials

Description of Additional Supplementary Files

Supplementary Data

Reporting Summary

## Data Availability

Data supporting the findings of this work are available within the paper and its Supplementary Data. The source data underlying Figs. 1b–e, 2a, b, d–m, 3a, b, e, g–j, 4a–g, 5a, c–f, and 6a–d are provided in Supplementary Data. The unedited/uncropped Western blot gels are provided in Supplementary Fig. 2. The other relevant data during the current study is available from the corresponding author upon request.

## Code availability

All custom code is available from the corresponding author upon reasonable request.

## References

[1] Klein BE (2007). Overview of epidemiologic studies of diabetic retinopathy. Ophthalmic Epidemiol..

[2] Saeedi P (2019). Global and regional diabetes prevalence estimates for 2019 and projections for 2030 and 2045: Results from the International Diabetes Federation Diabetes Atlas, 9(th) edition. Diabetes Res. Clin. Pract..

[3] Yau JW (2012). Global prevalence and major risk factors of diabetic retinopathy. Diabetes Care.

[4] Klaassen I, Van Noorden CJ, Schlingemann RO (2013). Molecular basis of the inner blood-retinal barrier and its breakdown in diabetic macular edema and other pathological conditions. Prog. Retin. Eye Res..

[5] Erickson KK, Sundstrom JM, Antonetti DA (2007). Vascular permeability in ocular disease and the role of tight junctions. Angiogenesis.

[6] Brownlee M (2001). Biochemistry and molecular cell biology of diabetic complications. Nature.

[7] Salzman J, Chen RE, Olsen MN, Wang PL, Brown PO (2013). Cell-type specific features of circular RNA expression. PLoS Genet..

[8] Li J (2015). Circular RNAs in cancer: novel insights into origins, properties, functions and implications. Am. J. Cancer Res..

[9] Ebert MS, Sharp PA (2010). MicroRNA sponges: progress and possibilities. RNA.

[10] Du WW (2017). Foxo3 circular RNA promotes cardiac senescence by modulating multiple factors associated with stress and senescence responses. Eur. Heart J..

[11] Shan K (2017). Circular noncoding RNA HIPK3 mediates retinal vascular dysfunction in diabetes mellitus. Circulation.

[12] Wei CY (2020). Circular RNA circ_0020710 drives tumor progression and immune evasion by regulating the miR-370-3p/CXCL12 axis in melanoma. Mol. Cancer.

[13] Peng F (2020). circRNA_010383 acts as a sponge for miR-135a and its downregulated expression contributes to renal fibrosis in diabetic nephropathy. Diabetes.

[14] Glazar P, Papavasileiou P, Rajewsky N (2014). circBase: a database for circular RNAs. RNA.

[15] Hansen TB (2013). Natural RNA circles function as efficient microRNA sponges. Nature.

[16] Azad, T., Ghahremani, M. & Yang, X. The role of YAP and TAZ in angiogenesis and vascular mimicry. *Cells* **8**. 10.3390/cells8050407 (2019). 10.3390/cells8050407 PMC6562567 31052445

[17] El Ouarrat D (2020). TAZ is a negative regulator of PPARgamma activity in adipocytes and TAZ deletion improves insulin sensitivity and glucose tolerance. Cell Metab..

[18] Memczak S (2013). Circular RNAs are a large class of animal RNAs with regulatory potency. Nature.

[19] Suganya N, Bhakkiyalakshmi E, Sarada DV, Ramkumar KM (2016). Reversibility of endothelial dysfunction in diabetes: role of polyphenols. Br. J. Nutr..

[20] Wang H, Wang AX, Aylor K, Barrett EJ (2013). Nitric oxide directly promotes vascular endothelial insulin transport. Diabetes.

[21] Wang Y (2019). Exosomal circHIPK3 released from hypoxia-pretreated cardiomyocytes regulates oxidative damage in cardiac microvascular endothelial cells via the miR-29a/IGF-1 pathway. Oxid. Med. Cell. Longev..

[22] Pei X, Ye S, Jin G, Yu Y (2018). Overexpression of circRNA-001175 promotes proliferation and angiogenesis and inhibits apoptosis of the human umbilical vein endothelial cells (HUVECs) induced by high glucose. Int. J. Clin. Exp. Pathol..

[23] Basha B, Samuel SM, Triggle CR, Ding H (2012). Endothelial dysfunction in diabetes mellitus: possible involvement of endoplasmic reticulum stress?. Exp. Diabetes Res..

[24] Bucciarelli LG (2009). Inflammatory stress in primary venous and aortic endothelial cells of type 1 diabetic mice. Diab. Vasc. Dis. Res..

[25] Ambros V (2004). The functions of animal microRNAs. Nature.

[26] Piccolo S, Dupont S, Cordenonsi M (2014). The biology of YAP/TAZ: hippo signaling and beyond. Physiol. Rev..

[27] Yu FX, Zhao B, Guan KL (2015). Hippo pathway in organ size control, tissue homeostasis, and cancer. Cell.

[28] Kim J (2017). YAP/TAZ regulates sprouting angiogenesis and vascular barrier maturation. J. Clin. Investig..

[29] Wang X (2017). YAP/TAZ orchestrate VEGF signaling during developmental angiogenesis. Dev. Cell.

[30] Puavilai G, Chanprasertyotin S, Sriphrapradaeng A (1999). Diagnostic criteria for diabetes mellitus and other categories of glucose intolerance: 1997 criteria by the Expert Committee on the Diagnosis and Classification of Diabetes Mellitus (ADA), 1998 WHO consultation criteria, and 1985 WHO criteria. World Health Organization. Diabetes Res. Clin. Pract..

