# Supplementary material for: Circular RNA RSU1 promotes retinal vascular dysfunction by regulating miR-345-3p/TAZ
Source: Commun Biol. 2023 Jul 13;6:719. doi: 10.1038/s42003-023-05064-x (PMC10344963; doi:10.1038/s42003-023-05064-x)

Supplementary Tables

Supplementary Table 1. Primers used for qRT-PCR

Genes	Forward (5'-3')	Reverse (5'-3')
circRSU1	TCGCTGCCTAAGGAAATCGG	ATGGCTGAGGACCAGTTGTG
miR-345-3p	TGGTTATTAGCCCTGAACGAG	AGTGCGTGTCGTGGAGTCG
TAZ	TGCTACAGTGTCCCCACAAC	GAAACGGGTCTGTTGGGGAT
VEGF	GGGCAGAATCATCACGAAGT	TGGTGATGTTGGACTCCTCA
β-actin	GAGAAAATCTGGCACCACACC	GGATAGCACAGCCTGGATAGCAA
U6	CTCGCTTCGGCAGCACA	AACGCTTCACGAATTTGCGT
circRSU1 Convergent	GAGCTTACCCAGCTTAAAGAGC	GGCACCATTGTTAGCTTGTTATG
circRSU1 Divergent	CATGAACAGGCTGAACACTTTG	TCAGGTAGAAGAAGTTTCCAGG
GAPDH Convergent	ATGGCCTCCAAGGAGTAAATG	AGGTCAATGAAGGGGTCATTG
GAPDH Divergent	AGAAGGCTGGGGCTCATTTG	GCAGGAGGCATTGCTGATGAT

Supplementary Figures

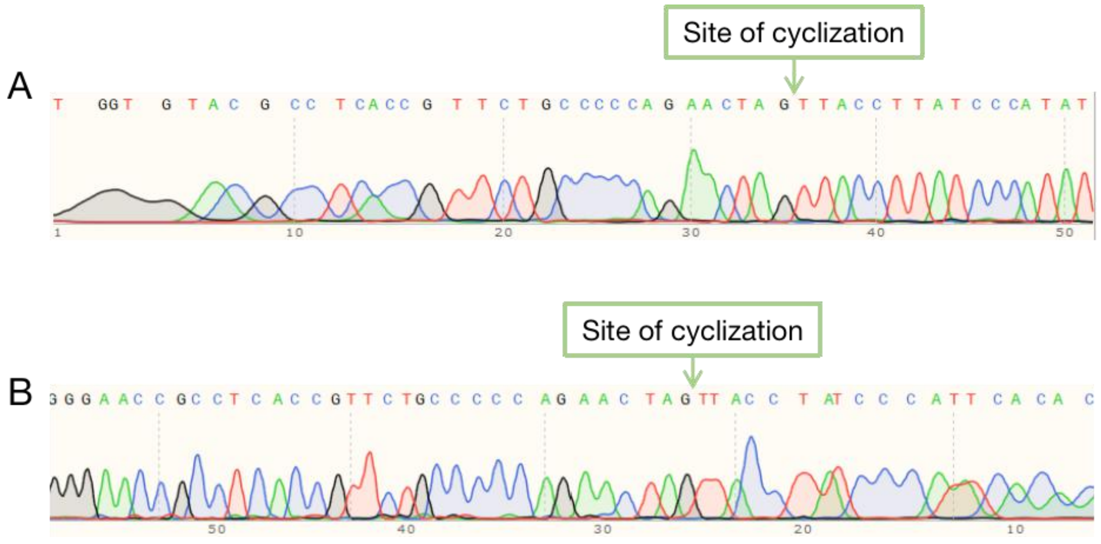

Supplementary Figure 1. DNA sequencing results of circRSU1.

The cyclization site sequence of curcR in circBase is GCCCCCAGAACTAGTTACCTTATCCCAT.

Supplementary Figure 2. Unedited/uncropped Western blot gels

a

Figure 2K

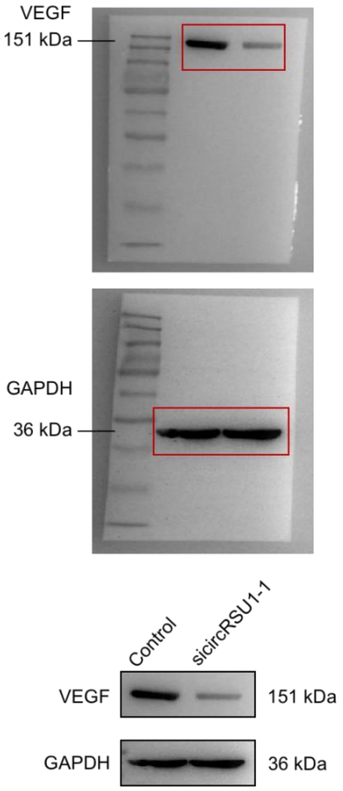

b

Figure 2L

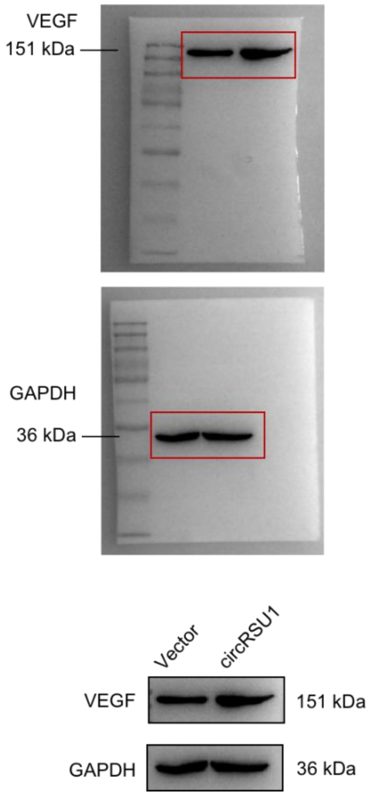

d

Figure 3H

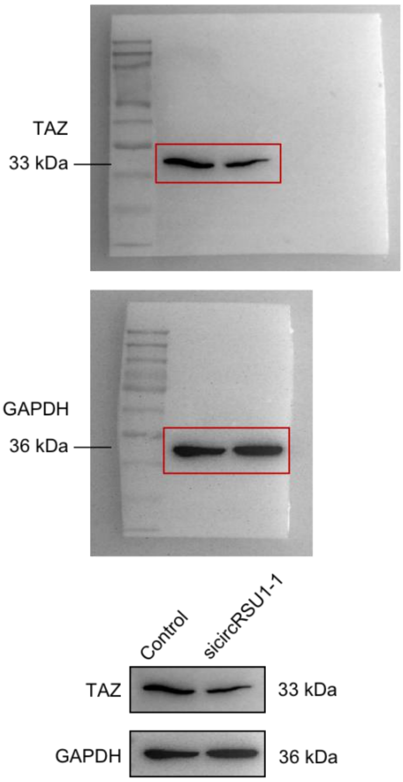

c

Figure 3A

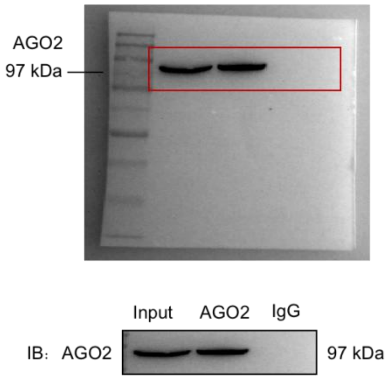

e

Figure 3I

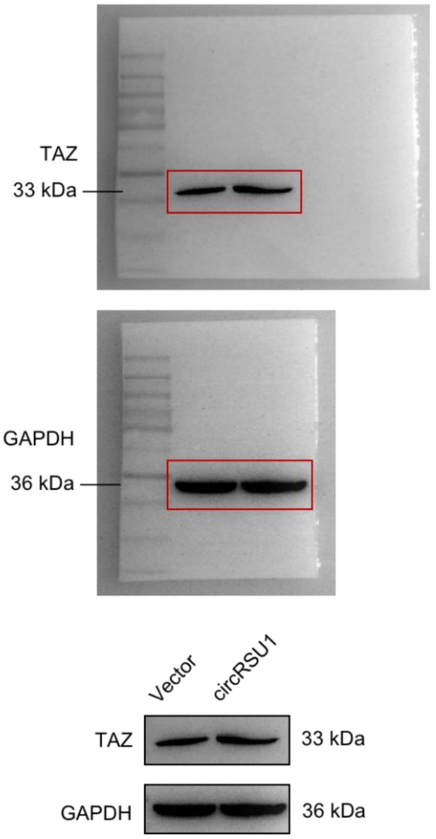

f

Figure 3J

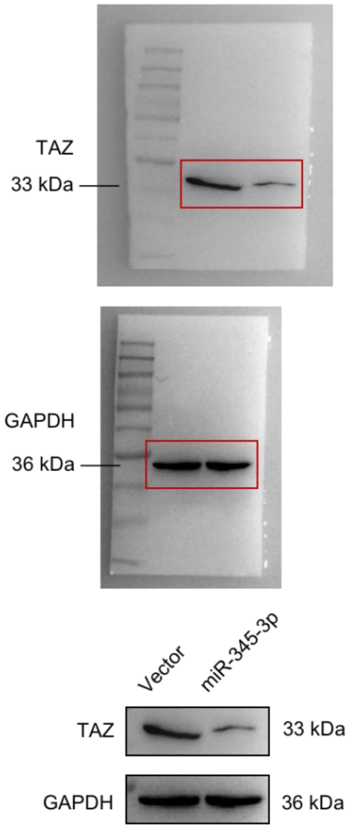

g

Figure 4G

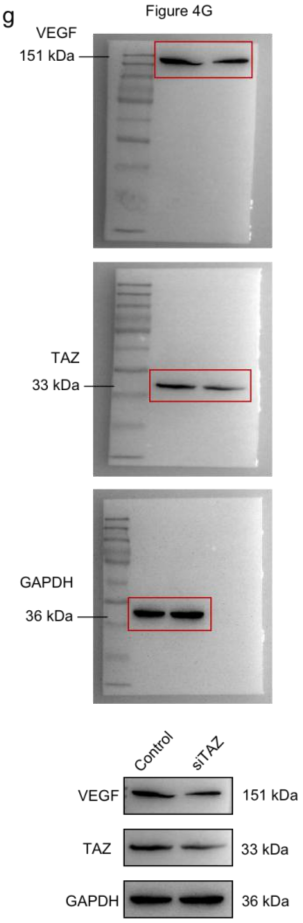

h

Figure 5F

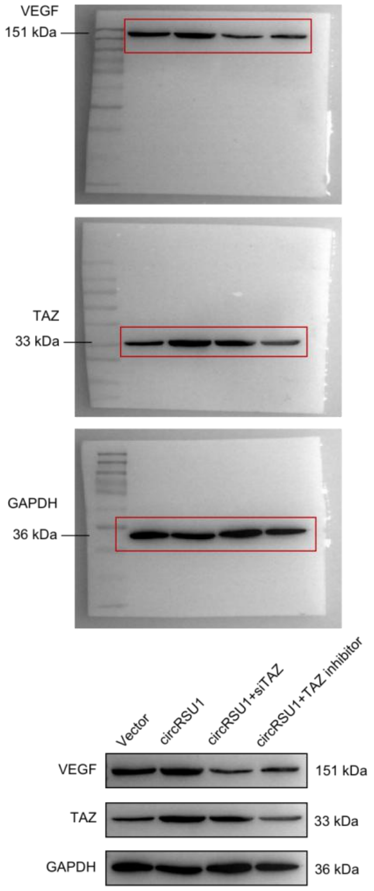

Supplement: Supplementary file 1 — Supplementary materials [file 42003_2023_5064_MOESM1_ESM.pdf]
