# Supplementary material for: Circular RNA RSU1 promotes retinal vascular dysfunction by regulating miR-345-3p/TAZ
Source: Commun Biol. 2023 Jul 13;6:719. doi: 10.1038/s42003-023-05064-x (PMC10344963; doi:10.1038/s42003-023-05064-x)
Supplement: Supplementary file 2 — Description of Additional Supplementary Files [file 42003_2023_5064_MOESM2_ESM.pdf]

Description of Additional Supplementary Files

File name: Supplementary Data

Description: The source data underlying Figs. 1b-e, 2a-b, 2d-m, 3a-b, 3e, 3g-j, 4a-g, 5a, 5c-f, and 6a-d.
