# Supplementary material for: Circular RNA RSU1 promotes retinal vascular dysfunction by regulating miR-345-3p/TAZ
Source: Commun Biol. 2023 Jul 13;6:719. doi: 10.1038/s42003-023-05064-x (PMC10344963; doi:10.1038/s42003-023-05064-x)
Supplement: Supplementary file 4 — Reporting Summary [file 42003_2023_5064_MOESM4_ESM.pdf]

nature portfolio

Corresponding author(s): Professor Ke Hu

Last updated by author(s): Jun 12, 2023

Reporting Summary

Nature Portfolio wishes to improve the reproducibility of the work that we publish. This form provides structure for consistency and transparency in reporting. For further information on Nature Portfolio policies, see our Editorial Policies and the Editorial Policy Checklist.

Statistics

For all statistical analyses, confirm that the following items are present in the figure legend, table legend, main text, or Methods section.

n/a	Confirmed	
☐	☒	The exact sample size (*n*) for each experimental group/condition, given as a discrete number and unit of measurement
☐	☒	A statement on whether measurements were taken from distinct samples or whether the same sample was measured repeatedly
☐	☒	The statistical test(s) used AND whether they are one- or two-sided<br>*Only common tests should be described solely by name; describe more complex techniques in the Methods section.*
☐	☒	A description of all covariates tested
☒	☐	A description of any assumptions or corrections, such as tests of normality and adjustment for multiple comparisons
☐	☒	A full description of the statistical parameters including central tendency (e.g. means) or other basic estimates (e.g. regression coefficient) AND variation (e.g. standard deviation) or associated estimates of uncertainty (e.g. confidence intervals)
☐	☒	For null hypothesis testing, the test statistic (e.g. *F*, *t*, *r*) with confidence intervals, effect sizes, degrees of freedom and *P* value noted<br>*Give P values as exact values whenever suitable.*
☒	☐	For Bayesian analysis, information on the choice of priors and Markov chain Monte Carlo settings
☐	☒	For hierarchical and complex designs, identification of the appropriate level for tests and full reporting of outcomes
☒	☐	Estimates of effect sizes (e.g. Cohen's *d*, Pearson's *r*), indicating how they were calculated

Our web collection on statistics for biologists contains articles on many of the points above.

Software and code

Policy information about availability of computer code

Data collection	Electronic Medical Record System version 4.5 and THIS version 4.0 (both from Winning Health Technology Group)
Data analysis	SPSS version 23.0 and Prism version 8

For manuscripts utilizing custom algorithms or software that are central to the research but not yet described in published literature, software must be made available to editors and reviewers. We strongly encourage code deposition in a community repository (e.g. GitHub). See the Nature Portfolio guidelines for submitting code & software for further information.

Data

Policy information about availability of data

All manuscripts must include a data availability statement. This statement should provide the following information, where applicable:

- Accession codes, unique identifiers, or web links for publicly available datasets
- A description of any restrictions on data availability
- For clinical datasets or third party data, please ensure that the statement adheres to our policy

Data supporting the findings of this study are available within the article and its supplementary material.

Human research participants

Policy information about studies involving human research participants and Sex and Gender in Research.

Reporting on sex and gender	Demographic data of all patients included sex and gender were analyzed. Among these patients, the ratio of Male/Female was 26/27 in DM group and 23/27 in DR group.
Population characteristics	A total of 103 patients with type 2 diabetes mellitus were recruited.
Recruitment	A cohort of 103 patients with type 2 diabetes were recruited from the First Affiliated Hospital of Chongqing Medical University, between March 2019 and March 2020, according to the guidelines described by the American Diabetes Association. All patients underwent a complete ophthalmological examination, including corrected slit-lamp microscopic examination fundoscopic examination, and fluorescence angiography. Patients with a history of rheumatic disease and inflammatory disease were excluded from the study.
Ethics oversight	the Ethics Committee of the First Affiliated Hospital of Chongqing Medical University

Note that full information on the approval of the study protocol must also be provided in the manuscript.

Field-specific reporting

Please select the one below that is the best fit for your research. If you are not sure, read the appropriate sections before making your selection.

☒ Life sciences ☐ Behavioural & social sciences ☐ Ecological, evolutionary & environmental sciences

For a reference copy of the document with all sections, see nature.com/documents/nr-reporting-summary-flat.pdf

Life sciences study design

All studies must disclose on these points even when the disclosure is negative.

Sample size	A total of 103 patients with type 2 diabetes mellitus. The Male Sprague-Dawley rats (80-100g) were used as a type 2 DM animal model. Intravitreal injection of sicircRSU1-1 (n = 18 eyes), scramble siRNA (n = 18 eyes) and miR-345-3p antagomir (n = 18 eyes) were performed in vivo experiments.
Data exclusions	No data were excluded from the analyses.
Replication	All of the data were obtained from three independent experiments.
Randomization	Participants were divided into two groups depending on the diagnosis. Animals were randomly averagely divided into different groups.
Blinding	The investigators were blinded to group allocation during data collection.

Reporting for specific materials, systems and methods

We require information from authors about some types of materials, experimental systems and methods used in many studies. Here, indicate whether each material, system or method listed is relevant to your study. If you are not sure if a list item applies to your research, read the appropriate section before selecting a response.

Materials & experimental systems

n/a	Involved in the study	
☐	☒	Antibodies
☐	☒	Eukaryotic cell lines
☒	☐	Palaeontology and archaeology
☐	☒	Animals and other organisms
☐	☒	Clinical data
☒	☐	Dual use research of concern

Methods

n/a	Involved in the study	
☒	☐	ChIP-seq
☒	☐	Flow cytometry
☒	☐	MRI-based neuroimaging

Antibodies

Antibodies used	1. VEGF receptor 1 (ab32152, Abcam), 2. GAPDH (M00227-5, BOSTER), 3. TAZ (ab224239, Abcam), 4. Ago2 (ab186733, Abcam) 5. secondary antibody: rabbit anti-rat IgG antibody (ab102248, Abcam).
Validation	Western blot (Beyotime).

Eukaryotic cell lines

Policy information about cell lines and Sex and Gender in Research

Cell line source(s)	The primary human retinal vascular endothelial cells (HRVECs) were purchased from Cell Systems (Kirkland, WA).
Authentication	VIII factor related antigen-antibody DAB staining.
Mycoplasma contamination	All cell lines tested negative for mycoplasma contamination.
Commonly misidentified lines (See ICLAC register)	HRVECs for vitro experiments.

Animals and other research organisms

Policy information about studies involving animals; ARRIVE guidelines recommended for reporting animal research, and Sex and Gender in Research

Laboratory animals	Male Sprague-Dawley rats (80-100g) were obtained from SLAC Laboratory Animal Co., Ltd (Shanghai, China).
Wild animals	The study did not involve wild animals.
Reporting on sex	Male rats were obtained for the study.
Field-collected samples	All the animals were kept on a 12-h light/dark cycle.
Ethics oversight	the Animal Ethics Committee of the First Affiliated Hospital of Chongqing Medical University

Note that full information on the approval of the study protocol must also be provided in the manuscript.

Clinical data

Policy information about clinical studies

All manuscripts should comply with the ICMJE guidelines for publication of clinical research and a completed CONSORT checklist must be included with all submissions.

Clinical trial registration	81900887 from the Ethics Committee of the First Affiliated Hospital of Chongqing Medical University
Study protocol	The plasma samples of all patients were gathered from blood, which was classified into diabetes mellitus participants without DR (n=53) and DR group (n=50). Medical files of all patients were reviewed by trained certified doctors to collect relevant medical information. CircRNAs and serum levels of VEGF were measured in this study.
Data collection	A cohort of 103 patients with type 2 diabetes were recruited from the First Affiliated Hospital of Chongqing Medical University, between March 2019 and March 2020. Demographic data of all patients included gender, age, body mass index, diabetes duration, family history, fasting glucose, HbA1c, circRSU1 and VEGF.
Outcomes	Main outcome: circRSU1 and VEGF. Secondary outcome: gender, age, body mass index, diabetes duration, family history, fasting glucose and HbA1c.
